# Racial/ethnic differences in the associations between social support and cardiovascular morbidity and mortality in the Multi-Ethnic Study of Atherosclerosis (MESA)

**DOI:** 10.1186/s12889-024-21141-0

**Published:** 2025-01-16

**Authors:** Jeanean B. Naqvi, Taynara Formagini, Matthew A. Allison, Namratha R. Kandula, Jee Won Park, Britta A. Larsen

**Affiliations:** 1 Department of Family Medicine, UC San Diego, La Jolla, CA USA; 2 Department of Preventive Medicine, Division of General Internal Medicine and Geriatrics, Department of Medicine, Northwestern University Feinberg School of Medicine, Chicago, IL USA; 3 Department of Epidemiology, University of Delaware, Newark, DE USA; 4 Herbert Wertheim School of Public Health & Human Longevity Science, UC San Diego, La Jolla, CA USA

**Keywords:** Social support, Race, Ethnicity, Culture, Cardiovascular morbidity, Cardiovascular mortality

## Abstract

**Background:**

Despite the established link between social support and cardiovascular disease (CVD) outcomes, few studies have examined racial/ethnic variation in these associations. This study utilized data from the Multi-Ethnic Study of Atherosclerosis (MESA) to investigate racial/ethnic differences in perceived social support and in the link between support and incident hard CVD events and mortality.

**Method:**

Participants (*N* = 6,814) were 45–84 years of age who identified as White, Black, Hispanic/Latino, or Chinese without known clinical CVD at baseline (2000–2002). Racial/ethnic differences in perceived support (overall, emotional, informational, and instrumental) were tested using multiple regression with adjustments for demographic, socioeconomic, lifestyle/psychosocial, and clinical risk factors, and immigration history. Racial/ethnic differences in the association between perceived support and incident CVD events or mortality were tested using Cox proportional hazards models with progressive adjustments for the same covariates.

**Results:**

At baseline, the mean age was 62.15 years (SD = 10.23); 38.5% identified as White, 27.8% as Black, 22.0% as Hispanic/Latino, and 11.8% as Chinese. Black and Hispanic/Latino participants reported higher levels of overall support, emotional support, and informational support than White participants (*p*’s < 0.05). Chinese participants reported less informational support (*p* = .010) than White participants. Higher informational support was associated with decreased risk for hard CVD events. This association did not differ by race/ethnic group.

**Conclusion:**

Despite racial/ethnic differences in perceptions of support, perceived informational support was protective against CVD for participants of all racial/ethnic backgrounds.

## Introduction

Systematic reviews show that a lack of social support is associated with the onset, prevalence, and progression of coronary heart disease (CHD) [1, 2], as well as cardiovascular mortality among those with preexisting CHD [2]. In contrast, higher levels of social support have a protective association with cardiovascular disease (CVD)—for example, higher emotional support was associated with lower risk of incident hard CVD events [3]. Perceived support, in particular, may be associated with cardiovascular health likely because it represents the perception of a “safety net” in times of need, contributing to positive affective and cognitive states and buffering against daily stressors that activate cardiovascular reactivity and inflammation [4, 5]. 

One limitation of this prior work is the assumption that social support plays the same role in cardiovascular health regardless of racial/ethnic background [6]. A burgeoning literature suggests that cultural background shapes individuals’ expectations of their relationship interactions, which may explain observed racial/ethnic differences in social support and its links to health [7]. For example, some studies show that compared to European Americans, Asian Americans appear to seek social support less and perceive it to be less helpful in reducing stress [8, 9]. Other studies suggest that Asian Americans find support more effective when it is unsolicited [10], perceived to be mutual or reciprocal [11], or received without needing to discuss the stressor explicitly [12]. In addition, some studies show higher levels of social support among Hispanic Americans compared to European Americans [13], especially regarding emotional support [14, 15]. Research comparing social support among Black and White individuals has been equivocal, with some studies finding higher support among White individuals [16, 17] and others finding higher support among Black individuals [18, 19]. Though inconsistent, these previous findings suggest there may be racial/ethnic differences in levels and types of social support between these groups.

Cultural background may also shape racial/ethnic differences in the associations between perceived support and cardiovascular outcomes. Both Hispanic/Latino and Asian cultural contexts are broadly collectivistic, viewing the self as interdependent with family members and prioritizing family needs over personal goals [7]. Though a meta-analysis found that Black individuals report more individualistic beliefs than White individuals [20], African American and Black Caribbean communities have a history of communalism and strong familial ties [21, 22]. These collectivistic values may reflect traditional cultural values from Asia, Latin America, Africa, and the Caribbean, as well as the need to develop strong support networks amidst pervasive racial/ethnic discrimination and socioeconomic struggle in the U.S [21–23]. Indeed, Asian, Hispanic, and Black individuals have a high proportion of kin in their networks [23–25]. However, these cultures also vary in important ways—for example, a key difference between Latino and Asian cultural values is that emotional expressiveness is more valued in Latino cultural contexts than in Asian cultural contexts [7], which may result in racial/ethnic differences in the link between emotional support and cardiovascular health. Despite both the shared and the distinct cultural factors among Asian, Latino, and Black cultural contexts that likely influence the social support process, little research has examined whether different types of social support might have a more beneficial impact on cardiovascular health among these racial/ethnic groups.

The purpose of this analysis was to examine racial/ethnic differences in types of perceived social support and in the associations between types of support and incident CVD events and mortality. We hypothesized that there would be racial/ethnic differences in overall levels of perceived support as well as types of perceived support: instrumental support (e.g., tangible assistance and provision of resources), emotional support (e.g., reassurance and emotional concern), and informational support (e.g., advice and suggestions). Similarly, we hypothesized that there would be racial/ethnic differences in the associations between overall levels or types of perceived support and incident CVD events and mortality.

## Method

### Study design and participants

The Multi-Ethnic Study of Atherosclerosis (MESA) is a multi-center longitudinal cohort study initiated to examine the prevalence, correlates, and progression of subclinical CVD. MESA included 6,814 men and women aged 45–84 years who identified as White, Black, Hispanic/Latino, or Chinese without known cardiovascular disease at the time of enrollment. Participants were recruited in 2000–2002 from six field centers (New York, NY; Baltimore, MD; Forsyth County, NC; St. Paul, MN; Chicago, IL; Los Angeles County, CA). The study received approval from the institutional review boards at all participating centers for all study visits (Columbia University; Johns Hopkins University; Wake Forest University; Northwestern University; University of California, Los Angeles; University of Minnesota), and informed consent was obtained from all participants. Details of the MESA recruitment and study protocol have been published elsewhere [26]. 

After the baseline visit, participants were invited to participate in five additional examinations: visit 2 (2002–2004), visit 3 (2004–2005), visit 4 (2005–2007), visit 5 (2010–2012), and visit 6 (2016–2018). Examinations were used to gather health-related data and monitor for incident cardiovascular events and mortality. Participants were censored at the time of last follow-up completed, or December 31, 2019, if the participant completed a follow-up on or after December 31, 2019. Our analytic sample included all baseline MESA participants with complete data for the exposure and outcome variables.

### Study variables

At baseline, participants self-identified as White, Black, Hispanic/Latino, or Chinese. Covariates were included based on conceptual understanding and to align closely with a prior MESA study examining racial/ethnic differences in CVD mortality [27]. Variables were obtained from interview-administered questionnaires and physical measurements at baseline. We included the following variables as covariates in the analyses: (1) demographic factors, including age (continuous variable) and gender; (2) sociodemographic factors, including educational level, household income level, and health insurance status, (3) lifestyle/psychosocial factors, including intentional exercise (metabolic equivalent minutes/week; continuous variable), diet quality (poor, intermediate, or ideal, as categorized in Life’s Simple 7 [28], smoking status (never, former, or current), smoking pack-years, alcohol use (never, former, or current), lifetime perceived discrimination (continuous variable), and chronic stress (burden; continuous variable); (4) clinical risk factors (all continuous variables unless otherwise specified), including body mass index (BMI), total cholesterol, high-density lipoprotein cholesterol, triglycerides, statin medication use, systolic and diastolic blood pressure, hypertension medication use and diabetes medication use (yes/no, with “don’t know” recoded as “no”), and fasting glucose; and (5) immigration history, in which participants were categorized as U.S.-born, immigrated to the U.S. <30 years ago, and immigrated to the U.S. ≥30 years ago using participants’ country of birth and length of time living in the U.S.

Perceived social support was measured at baseline using the ENRICHD Social Support Inventory (ESSI) [29]. All six items were measured on a 5-point scale (1 = none of the time; 5 = all of the time). Overall social support was measured by calculating the average of all six items (α = 0.88). Perceived emotional support was assessed by averaging the responses from the items regarding having someone available to listen, show love and affection, provide emotional support, and having sufficient contact with someone to confide in. Perceived informational support was measured with the item “someone available to give you advice.” Perceived instrumental support was measured using the item “someone available to help with daily chores.” Perceived support variables were moderately to strongly correlated with each other, ranging from 0.34 to 0.96.

Incident hard CVD events included definite and probable myocardial infarction, resuscitated cardiac arrest, coronary heart disease (CHD) death, stroke, and stroke death at any of the follow-up visits (visits 2 to 6). CVD mortality was defined as death attributed to atherosclerotic coronary heart disease, stroke, atherosclerotic disease other than coronary disease, or other CVD at any follow-up visit (visits 2 to 6). Trained physicians adjudicated self-reported diagnoses through death certificates and medical record reviews. Reviewers categorized myocardial infarction as definite, probable, or absent based on a combination of symptoms, ECG abnormalities, and cardiac biomarkers. Fatal coronary heart disease was established by myocardial infarction within 28 days before death, chest pain within 72 h before death, or a history of coronary heart disease, along with the absence of known nonatherosclerotic or noncardiac causes of death. Stroke was confirmed through neurological deficits lasting 24 h or until death, and if less than 24 h, by a brain lesion observed through imaging. Patients with neurological deficits from brain trauma, tumor, infection, or other nonvascular causes were classified as “no stroke.” The “stroke” category encompassed all types, both fatal and nonfatal, including hemorrhagic strokes.

### Data analysis

Descriptive statistics include sample size and percentages for categorical variables and means and standard deviations for continuous variables, without imputation for missing values and stratified by race/ethnicity (Table 1). To examine racial/ethnic differences in perceived social support, we conducted multiple linear regression and adjusted for demographic factors, socioeconomic factors, lifestyle/psychosocial factors, clinical risk factors, and immigration history. Table 2 reports the unadjusted means, the estimated marginal means, and the *p*-values from the multiple regression models, which indicate whether there was a significant difference in perceived support between White participants (the reference group) and the specified racial/ethnic group.

To examine racial/ethnic differences in the link between perceived support and incident hard CVD events or CVD mortality, we used Cox proportional hazards models. Main effects models (no interaction between race/ethnicity and the specified support variable) and interaction models (multiplicative interaction between race/ethnicity and the specified support variable) were conducted. We compared the goodness of fit of each main effects model versus the interaction model using a likelihood ratio test. Hazard Ratios (HR) with 95% CIs, along with the *p*-values from the likelihood ratio tests, are reported in Tables 3 and 4. After testing an unadjusted model, progressive adjustments of groups of covariates were included to control for their potential confounding effect in the association between the exposure and outcome variables in the following order: (1) demographic factors, (2) socioeconomic factors, (3) lifestyle/psychosocial factors, (4) clinical risk factors, and (5) immigration history. We checked the proportional hazards assumption for all model predictors using scaled Schoenfeld residuals. Significant results were found for intentional exercise, systolic blood pressure, and diastolic blood pressure for the Cox models examining incident hard CVD events, as well as intentional exercise and chronic stress for the Cox models examining CVD mortality. For each covariate with significant results, we examined plots of the scaled Schoenfield residuals against time. The departure from proportional hazards was not visible upon visual inspection. A sensitivity analysis excluding these covariates from the models did not alter the results substantially.

Missing covariate data were imputed for the primary analyses using multiple imputation by chained equations (MICE), which estimates values based on variable relationships by creating multiple imputed datasets where missing values are predicted [31]. No more than 5% of data were missing from any imputed variables. Participants who were missing data for at least one of the exposure or outcome variables (*n* = 73) were older, were less likely to have health insurance, reported lower levels of intentional exercise, and had higher levels of diabetes medication use than participants without missing data. All analyses were conducted using R version 4.2.2, and the Benjamini-Hochberg procedure was used to correct all *p*-values for multiple comparisons [30] with statistical significance being set at *p* < .05.

## Results

At baseline, MESA participants (*N* = 6,814) were 47.2% male and 62.15 years of age on average (SD = 10.23), with 38.5% identifying as White, 27.8% as Black, 22.0% as Hispanic/Latino, and 11.8% as Chinese. Participants had a median household income of $35,000 to $39,999, and 35.2% had received a bachelor’s or graduate degree. Baseline characteristics of the study population, stratified by race and ethnicity, are provided in Table 1. Differences in socioeconomic, lifestyle, psychosocial, and clinical factors were observed by race/ethnicity (Table 1) and are discussed in more detail in other publications [27]. 

**Table 1:** Baseline characteristics of MESA participants by race/ethnicity, 2000–2002

	White	Black	Chinese	Hispanic/Latino
	*N* = 2,622	*N* = 1,892	*N* = 804	*N* = 1,496
**Age**,** M (SD)**	62.60 (10.25)	62.15 (10.05)	62.34 (10.33)	61.27 (10.34)
**Gender (male)**, ***n*(%)**	1,260 (48.1%)	842 (44.5%)	390 (48.5%)	721 (48.2%)
**Education level (highest degree)**, ***n*(%)**				
No degree	129 (4.9%)	229 (12.2%)	199 (24.8%)	668 (44.7%)
High school or associate’s degree	1,188 (45.4%)	1,013 (53.9%)	292 (36.4%)	680 (45.5%)
Bachelor’s degree	581 (22.2%)	325 (17.3%)	182 (22.7%)	83 (5.5%)
Graduate degree	716 (27.4%)	311 (16.6%)	130 (16.2%)	65 (4.3%)
**Household income**, ***n*(%)**				
< $25,000	413 (16.2%)	530 (30.6%)	395 (49.5%)	722 (49.5%)
$25,000 - $49,999	681 (26.7%)	558 (32.2%)	175 (21.9%)	478 (32.7%)
$50,000 - $99,999	836 (32.8%)	500 (28.9%)	148 (18.5%)	225 (15.4%)
$100,000+	621 (24.3%)	144 (8.3%)	80 (10.0%)	35 (2.4%)
**Health insurance**, ***n*(%)**	2,545 (97.3%)	1,762 (93.8%)	649 (80.8%)	1,227 (82.0%)
**Intentional exercise (metabolic equivalent min/wk)**,** M (SD)**	1,686.63 (2,300.71)	1,712.10 (2,784.83)	1,147.93 (1,517.59)	1,335.87 (2,109.64)
**Diet (poor)**, ***n*(%)**	1,606 (63.1%)	1,096 (62.6%)	248 (30.8%)	958 (66.9%)
**Smoking status**, ***n*(%)**				
Never	1,158 (44.3%)	848 (45.2%)	605 (75.3%)	807 (53.9%)
Former	1,156 (44.2%)	692 (36.8%)	153 (19.1%)	486 (32.5%)
Current	301 (11.5%)	338 (18.0%)	45 (5.6%)	203 (13.6%)
**Pack-years (among smokers)**,** M (SD)**	26.98 (27.93)	22.03 (21.96)	20.04 (22.73)	16.91 (20.95)
**Alcohol status**, ***n*(%)**				
Never	245 (9.4%)	325 (17.4%)	432 (54.0%)	388 (26.0%)
Former	484 (18.6%)	620 (33.2%)	118 (14.8%)	402 (26.9%)
Current	1,869 (71.9%)	925 (49.5%)	250 (31.3%)	705 (47.2%)
**Lifetime perceived discrimination**,** M (SD)**	0.57 (0.89)	1.22 (1.32)	0.30 (0.69)	0.67 (1.02)
**Chronic stress (burden)**,** M (SD)**	1.23 (1.18)	1.36 (1.25)	0.79 (1.07)	1.25 (1.20)
**Low-density lipoprotein cholesterol (mg/dl)**,** M (SD)**	117.05 (30.15)	116.47 (33.03)	115.09 (28.95)	119.54 (32.85)
**High-density lipoprotein cholesterol (mg/dl)**,** M (SD)**	52.24 (15.69)	52.42 (15.28)	49.53 (12.71)	47.65 (13.07)
**Total cholesterol (mg/dl)**,** M (SD)**	195.71 (35.13)	189.64 (36.27)	192.61 (31.78)	197.95 (37.45)
**Triglycerides (mg/dl)**,** M (SD)**	132.89 (90.21)	104.79 (68.58)	142.70 (84.72)	157.05 (101.08)
**Statin medication**, ***n*(%)**	436 (16.7%)	291 (15.4%)	103 (12.8%)	180 (12.0%)
**Systolic blood pressure (mmHg)**,** M (SD)**	123.49 (20.43)	131.68 (21.58)	124.57 (21.62)	126.68 (21.89)
**Diastolic blood pressure (mmHg)**,** M (SD)**	70.24 (9.97)	74.48 (10.21)	71.99 (10.34)	71.56 (10.12)
**Hypertension medication**, ***n*(%)**	868 (33.1%)	950 (50.3%)	231 (28.7%)	487 (32.6%)
**Diabetes medication**, ***n*(%)**	123 (4.7%)	263 (13.9%)	72 (9.0%)	230 (15.4%)
**Fasting glucose (mg/dL)**,** M (SD)**	91.41 (21.55)	100.04 (32.00)	98.95 (28.23)	103.63 (39.08)
**Body mass index (kg/m**^**2**^**)**,** M (SD)**	27.73 (5.06)	30.17 (5.88)	23.99 (3.30)	29.43 (5.10)
**Immigration history**, ***n*(%)**				
U.S. born	2,444 (94.9%)	1,712 (93.0%)	30 (4.0%)	466 (33.8%)
Immigrant to U.S. < 30 y ago	32 (1.2%)	69 (3.8%)	576 (76.7%)	416 (30.2%)
Immigrant to U.S. ≥ 30 y ago	100 (3.9%)	59 (3.2%)	145 (19.3%)	495 (35.9%)

Note: This table presents descriptive statistics for the study sample prior to multiple imputation. Missing data are omitted

Table 2 presents the results testing racial/ethnic differences in social support. In the adjusted models, Black and Hispanic/Latino participants reported higher average levels of overall support, emotional support, and informational support than White participants (*p*’s < 0.05). Chinese participants reported lower average levels of informational support (*p* = .010) than White participants.

**Table 2:** Racial/ethnic differences in perceived social support

	Unadjusted Model		Adjusted Model	
	Estimated Mean	*p*	Estimated Mean	*p*
**Overall Support** (*n* = 6,768)				
White	4.02	—	4.00	—
Black	4.05	0.39	4.15	**< 0.001**
Chinese	3.97	0.22	3.93	0.22
Hispanic/Latino	4.04	0.56	4.12	**0.004**
**Emotional Support** (*n* = 6,771)				
White	4.18	—	4.14	—
Black	4.22	0.22	4.31	**< 0.001**
Chinese	4.09	**0.029**	4.06	0.14
Hispanic/Latino	4.19	0.68	4.27	**0.003**
**Informational Support** (*n* = 6,780)				
White	4.04	—	4.01	—
Black	4.16	**< 0.001**	4.22	**< 0.001**
Chinese	3.92	**0.013**	3.84	**0.010**
Hispanic/Latino	4.08	0.25	4.12	**0.023**
**Instrumental Support** (*n* = 6,782)				
White	3.38	—	3.41	—
Black	3.24	**0.008**	3.42	0.76
Chinese	3.55	**0.010**	3.52	0.22
Hispanic/Latino	3.39	0.75	3.50	0.17

The unadjusted model presents the unadjusted means

The adjusted model presents the estimated marginal means adjusted for: age, gender, education, income, health insurance, intentional exercise, diet, smoking status, smoking pack-years, alcohol status, lifetime perceived discrimination, lifetime chronic stress (burden), body mass index, total cholesterol, high-density lipoprotein cholesterol, triglycerides, statin medication use, systolic blood pressure, diastolic blood pressure, hypertension medication use, diabetes medication use, fasting glucose, and immigration history

Each *p*-value was corrected using the Benjamini-Hochberg procedure and indicates whether there was a significant difference in perceived support between White participants (the reference group) and the specified racial/ethnic group. The *n* represents the number of observations included in all models, which excludes missing observations for the specified social support variable

Over a median follow-up period of 18 years, a total of 874 participants experienced a hard CVD event, and 682 died from CVD. Table 3 presents the results testing racial/ethnic differences in the associations between different social support measures and incident hard CVD events. In the fully adjusted main effects models (Model 5), only informational support was significantly associated with decreased risk for incident hard CVD events after correcting using the Benjamini-Hochberg procedure (HR: 0.90; 95% CI: 0.82–0.98). Although effects for overall support (HR: 0.89; 95% CI: 0.81–0.97) and emotional support (HR: 0.90; 95% CI: 0.82–0.98) in the fully adjusted main effects models (Model 5) were no longer significantly associated with decreased risk for incident hard CVD events after the Benjamini-Hochberg procedure, their confidence intervals did not include 1. In models adjusting for demographics and socioeconomic status (Model 2), higher levels of overall support (HR: 0.90; 95% CI: 0.83–0.98) and emotional support (HR: 0.90; 95% CI: 0.83–0.98) were associated with decreased risk for incident hard CVD events, though these effects was no longer significant after adjusting for lifestyle and psychosocial factors (Model 3). None of the associations differed significantly by race/ethnic group.

Table 4 presents the results testing racial/ethnic differences in the associations between different social support measures and CVD mortality. In the fully adjusted main effects models (Model 5), effects for overall support (HR: 0.89; 95% CI: 0.81–0.99), emotional support (HR: 0.89; 95% CI: 0.81–0.99), and informational support (HR: 0.89; 95% CI: 0.81–0.98) were no longer significantly associated with decreased risk for CVD mortality after the Benjamini-Hochberg procedure; however, their confidence intervals did not include 1. None of the associations differed significantly by race/ethnic group.

**Table 3:** Hazard ratios for racial/ethnic differences in the associations between perceived support and incident hard cardiovascular disease events

	Model 0			Model 1			Model 2			Model 3			Model 4			Model 5	
	HR (95% CI)	*p*		HR (95% CI)	*p*		HR (95% CI)	*p*		HR (95% CI)	*p*		HR (95% CI)	*p*		HR (95% CI)	*p*
**Overall Support** (*n* = 6,741)	0.91 (0.84–0.99)			**0.88 (0.81–0.96)**			**0.90 (0.83–0.98)**			0.91 (0.83–0.99)			0.89 (0.81–0.97)			0.89 (0.81–0.97)	
**Race × Overall Support**		0.85			0.85			0.85			0.85			0.85			0.85
White	1.00 Ref			1.00 Ref			1.00 Ref			1.00 Ref			1.00 Ref			1.00 Ref	
Black	0.95 (0.77–1.17)			0.97 (0.79–1.20)			0.95 (0.77–1.18)			0.95 (0.77–1.17)			0.95 (0.77–1.17)			0.95 (0.77–1.17)	
Chinese	1.40 (0.94–2.08)			1.33 (0.90–1.97)			1.28 (0.87–1.89)			1.31 (0.89–1.95)			1.32 (0.89–1.95)			1.33 (0.90–1.96)	
Hispanic/Latino	0.98 (0.79–1.20)			0.99 (0.81–1.22)			0.97 (0.79–1.20)			0.97 (0.79–1.20)			0.98 (0.80–1.21)			0.98 (0.80–1.21)	
**Emotional Support** (*n* = 6,744)	0.91 (0.84–0.99)			**0.88 (0.81–0.96)**			**0.90 (0.83–0.98)**			0.91 (0.83–0.99)			0.90 (0.82–0.98)			0.90 (0.82–0.98)	
**Race × Emotional Support**		0.85			0.85			0.85			0.85			0.85			0.85
White	1.00 Ref			1.00 Ref			1.00 Ref			1.00 Ref			1.00 Ref			1.00 Ref	
Black	0.89 (0.72–1.10)			0.91 (0.74–1.12)			0.90 (0.73–1.11)			0.89 (0.72–1.10)			0.89 (0.72–1.10)			0.89 (0.72–1.10)	
Chinese	1.25 (0.86–1.83)			1.22 (0.84–1.78)			1.18 (0.81–1.72)			1.20 (0.82–1.76)			1.24 (0.85–1.80)			1.24 (0.85–1.80)	
Hispanic/Latino	0.93 (0.76–1.14)			0.94 (0.77–1.15)			0.93 (0.76–1.14)			0.93 (0.76–1.14)			0.95 (0.77–1.16)			0.95 (0.77–1.16)	
**Informational Support** (*n* = 6,753)	**0.92 (0.84-1.00)**			**0.90 (0.83–0.98)**			**0.91 (0.84–0.99)**			0.92 (0.85–1.01)			**0.90 (0.82–0.98)**			**0.90 (0.82–0.98)**	
**Race × Informational Support**		0.85			0.85			0.85			0.85			0.85			0.85
White	1.00 Ref			1.00 Ref			1.00 Ref			1.00 Ref			1.00 Ref			1.00 Ref	
Black	0.92 (0.74–1.15)			0.94 (0.76–1.17)			0.92 (0.74–1.15)			0.91 (0.74–1.14)			0.93 (0.74–1.15)			0.92 (0.74–1.15)	
Chinese	1.23 (0.86–1.76)			1.24 (0.87–1.77)			1.19 (0.83–1.70)			1.22 (0.85–1.75)			1.25 (0.87–1.80)			1.26 (0.88–1.81)	
Hispanic/Latino	0.94 (0.77–1.16)			0.96 (0.78–1.18)			0.95 (0.78–1.17)			0.95 (0.77–1.16)			0.96 (0.78–1.18)			0.96 (0.78–1.18)	
**Instrumental Support** (*n* = 6,755)	0.96 (0.88–1.05)			**0.92 (0.84-1.00)**			0.95 (0.87–1.04)			0.95 (0.87–1.04)			0.93 (0.85–1.02)			0.93 (0.85–1.02)	
**Race × Instrumental Support**		0.85			0.85			0.85			0.85			0.85			0.85
White	1.00 Ref			1.00 Ref			1.00 Ref			1.00 Ref			1.00 Ref			1.00 Ref	
Black	1.16 (0.93–1.43)			1.19 (0.96–1.48)			1.15 (0.93–1.43)			1.15 (0.93–1.42)			1.16 (0.94–1.44)			1.16 (0.94–1.44)	
Chinese	1.45 (1.02–2.07)			1.39 (0.98–1.97)			1.34 (0.94–1.89)			1.35 (0.95–1.92)			1.29 (0.91–1.83)			1.29 (0.91–1.84)	
Hispanic/Latino	1.16 (0.93–1.44)			1.19 (0.96–1.48)			1.16 (0.93–1.44)			1.16 (0.93–1.44)			1.12 (0.90–1.40)			1.12 (0.90–1.40)	

Model 0 presents the unadjusted results

Model 1 adjusts for demographics: age, gender

Model 2 adjusts for socioeconomic status: education, income, health insurance

Model 3 adjusts for lifestyle and psychosocial factors: intentional exercise, diet, smoking status, smoking pack-years, alcohol status, lifetime perceived discrimination, lifetime chronic stress (burden)

Model 4 adjusts for clinical risk factors: body mass index, total cholesterol, high-density lipoprotein cholesterol, triglycerides, statin medication use, systolic blood pressure, diastolic blood pressure, hypertension medication use, diabetes medication use, fasting glucose

Model 5 adjusts for immigration history

Results are reported for main effects models (no interaction term between race and each support variable) and interaction models (interaction term between race and each support variable). Each *p*-value was corrected using the Benjamini-Hochberg procedure and indicates whether the interaction model had a better fit to the data compared to the main effects model, as assessed via a likelihood ratio test. Statistically significant HRs after the Benjamini-Hochberg procedure are bolded. The *n* represents the number of observations included in all models, which excludes missing observations for the specified social support variable, hard CVD event, and/or time to hard CVD event

**Table 4:** Hazard ratios for racial/ethnic differences in the associations between perceived support and cardiovascular disease mortality

	Model 0			Model 1			Model 2			Model 3			Model 4			Model 5	
	HR (95% CI)	*p*		HR (95% CI)	*p*		HR (95% CI)	*p*		HR (95% CI)	*p*		HR (95% CI)	*p*		HR (95% CI)	*p*
**Overall Support** (*n* = 6,751)	0.91 (0.82-1.00)			0.87 (0.79–0.95)			0.88 (0.80–0.97)			0.90 (0.82-1.00)			0.89 (0.81–0.99)			0.89 (0.81–0.99)	
**Race × Overall Support**		0.60			0.60			0.60			0.60			0.60			0.60
White	1.00 Ref			1.00 Ref			1.00 Ref			1.00 Ref			1.00 Ref			1.00 Ref	
Black	0.98 (0.77–1.23)			1.01 (0.80–1.27)			0.99 (0.79–1.25)			0.99 (0.79–1.25)			0.98 (0.78–1.24)			0.98 (0.78–1.24)	
Chinese	1.40 (0.95–2.07)			1.32 (0.90–1.94)			1.26 (0.86–1.86)			1.29 (0.87–1.91)			1.27 (0.85–1.88)			1.28 (0.86–1.90)	
Hispanic/Latino	1.13 (0.88–1.44)			1.15 (0.91–1.47)			1.14 (0.89–1.45)			1.15 (0.90–1.46)			1.15 (0.91–1.47)			1.16 (0.91–1.47)	
**Emotional Support** (*n* = 6,754)	0.90 (0.82–0.99)			0.87 (0.79–0.95)			0.88 (0.80–0.96)			0.90 (0.82–0.99)			0.89 (0.81–0.99)			0.89 (0.81–0.99)	
**Race × Emotional Support**		0.60			0.60			0.60			0.60			0.60			0.60
White	1.00 Ref			1.00 Ref			1.00 Ref			1.00 Ref			1.00 Ref			1.00 Ref	
Black	0.99 (0.79–1.25)			1.02 (0.81–1.28)			1.01 (0.80–1.27)			1.01 (0.80–1.27)			1.00 (0.79–1.26)			1.00 (0.79–1.26)	
Chinese	1.32 (0.91–1.91)			1.27 (0.88–1.83)			1.22 (0.85–1.77)			1.23 (0.85–1.79)			1.22 (0.84–1.78)			1.23 (0.84–1.80)	
Hispanic/Latino	1.12 (0.88–1.42)			1.14 (0.90–1.44)			1.12 (0.89–1.42)			1.13 (0.89–1.43)			1.15 (0.91–1.45)			1.15 (0.91–1.45)	
**Informational Support** (*n* = 6,763)	0.89 (0.81–0.98)			0.88 (0.80–0.96)			0.88 (0.80–0.97)			0.90 (0.82–0.99)			0.89 (0.81–0.98)			0.89 (0.81–0.98)	
**Race × Informational Support**		0.60			0.60			0.60			0.60			0.60			0.60
White	1.00 Ref			1.00 Ref			1.00 Ref			1.00 Ref			1.00 Ref			1.00 Ref	
Black	0.89 (0.70–1.12)			0.91 (0.72–1.15)			0.90 (0.71–1.14)			0.89 (0.71–1.13)			0.90 (0.71–1.14)			0.90 (0.71–1.13)	
Chinese	1.13 (0.80–1.61)			1.15 (0.81–1.63)			1.12 (0.78–1.59)			1.14 (0.80–1.63)			1.14 (0.80–1.63)			1.15 (0.80–1.64)	
Hispanic/Latino	1.04 (0.81–1.33)			1.06 (0.83–1.35)			1.05 (0.83–1.35)			1.05 (0.82–1.35)			1.07 (0.84–1.37)			1.08 (0.84–1.38)	
**Instrumental Support** (*n* = 6,765)	0.98 (0.90–1.08)			0.94 (0.86–1.04)			0.96 (0.87–1.06)			0.98 (0.89–1.08)			0.96 (0.87–1.06)			0.96 (0.87–1.06)	
**Race × Instrumental Support**		0.60			0.60			0.60			0.60			0.60			0.60
White	1.00 Ref			1.00 Ref			1.00 Ref			1.00 Ref			1.00 Ref			1.00 Ref	
Black	1.02 (0.81–1.29)			1.05 (0.84–1.33)			1.03 (0.82–1.30)			1.01 (0.80–1.28)			1.02 (0.81–1.29)			1.02 (0.81–1.29)	
Chinese	1.36 (0.97–1.90)			1.30 (0.93–1.81)			1.24 (0.89–1.72)			1.25 (0.90–1.75)			1.22 (0.87–1.71)			1.23 (0.88–1.72)	
Hispanic/Latino	1.15 (0.90–1.49)			1.19 (0.92–1.53)			1.17 (0.91–1.50)			1.16 (0.90–1.49)			1.13 (0.88–1.46)			1.13 (0.88–1.46)	

Model 0 presents the unadjusted results

Model 1 adjusts for demographics: age, gender

Model 2 adjusts for socioeconomic status: education, income, health insurance

Model 3 adjusts for lifestyle and psychosocial factors: intentional exercise, diet, smoking status, smoking pack-years, alcohol status, lifetime perceived discrimination, lifetime chronic stress (burden)

Model 4 adjusts for clinical risk factors: body mass index, total cholesterol, high-density lipoprotein cholesterol, triglycerides, statin medication use, systolic blood pressure, diastolic blood pressure, hypertension medication use, diabetes medication use, fasting glucose

Model 5 adjusts for immigration history

Results are reported for main effects models (no interaction term between race and each support variable) and interaction models (interaction term between race and each support variable). Each *p*-value was corrected using the Benjamini-Hochberg procedure and indicates whether the interaction model had a better fit to the data compared to the main effects model, as assessed via a likelihood ratio test. Statistically significant HRs after the Benjamini-Hochberg procedure are bolded. The *n* represents the number of observations included in all models, which excludes missing observations for the specified social support variable, CVD death, and/or time to CVD death

## Discussion

Among participants in MESA, evidence from the current analysis supported our first hypothesis that there would be racial/ethnic differences in perceived support. After adjusting for covariates, Black and Hispanic/Latino participants reported higher overall support, emotional support, and informational support than White participants, whereas Chinese participants reported less informational support than White participants. However, and contrary to our second hypothesis, the associations between perceived support and incident hard CVD events or CVD mortality did not differ by racial/ethnic group. We found evidence that higher levels of informational support were protective against incident CVD events regardless of racial/ethnic group in this study.

A growing literature shows that cultural background influences perceptions and expectations of social support [7]. Within the current study, we observed more overall support, emotional support, and informational support among Hispanic/Latino participants than White participants, aligning with values such as familismo and simpatía that emphasize support and warmth within close relationships [7, 32, 33]. Second, Black participants also reported more overall support, emotional support, and informational support than White participants, which might be explained by communalism and close kinship ties in African American and Black Caribbean communities [21, 22]. Third, Chinese participants reported less informational support than White participants, which seems to dovetail with previous literature suggesting that Asian individuals seek social support less and perceive it to be less helpful in reducing stress than White individuals [8, 9]. Interestingly, the unadjusted models showed that White participants reported less instrumental support than Chinese participants, but more instrumental support than Black participants. These effects disappeared after adjusting for covariates, suggesting that other factors (e.g., socioeconomic status) may play an important role in instrumental support receipt. Overall, these findings underscore the importance of examining racial/ethnic differences in levels of overall support as well as type of support in more depth to better understand the cultural mechanisms that may explain these differences.

Though Asian, Hispanic/Latino, and Black cultural contexts emphasize strong family ties [7, 34], we did not observe racial/ethnic differences in the links between perceived support and incident CVD events or CVD mortality. This might reflect variations in unmeasured social factors associated with race/ethnicity, social support, and CVD, including healthcare quality, cultural practices and values, and neighborhood factors such as social cohesion and access to resources, all of which may have affected our ability to detect racial/ethnic differences in the link between social support and CVD outcomes. Alternatively, social support may mitigate the impact of socioeconomic status or discrimination on cardiovascular health in racial/ethnic minoritized populations, but these variables were controlled for within the current analysis. More research is needed to explore these associations.

When examining main effects for perceived support regardless of racial/ethnic group, prior research has established consistent inverse associations between social support and CVD morbidity and mortality [1, 2], and other MESA analyses have found an decreased risk of incident CVD with higher overall support [35] and higher emotional support [3]. However, the only consistent pattern of inverse association observed in the current analysis was between informational support and incident hard CVD events, which is surprising given that previous research has suggested that emotional support may be more beneficial for health than informational or instrumental support [36]. When others provide advice or assistance, this may communicate that others believe the individual is in need of help, which may lower the individuals’ self-efficacy and sense of control [37]. However, our results suggest possible benefits of the perceived availability of advice or suggestions from others.

Patterns of inverse association were also observed between overall support or emotional support and incident hard CVD events before adjusting for lifestyle and psychosocial factors, which suggests that these covariates may influence these associations. For example, individuals may experience encouragement from their social network around eating healthier or exercising more, leading to improved diet and exercise and ultimately lowering the risk for incident hard CVD events. Alternatively, a lack of overall support may partially account for higher levels of lifetime chronic stress, leading to increased risk for incident hard CVD events. Although overall support, emotional support, and informational support were not significantly associated with decreased risk for CVD mortality after the Benjamini-Hochberg procedure, there was weak evidence to corroborate previous findings given that the hazard ratio confidence intervals did not include 1.

There are limitations to this analysis. First, we were unable to account for competing risks, and they were treated as censoring events in our Cox proportional hazard models. This may have overestimated our findings from the Cox models. Second, only baseline measures of perceived support were included in the models, and informational and instrumental support were only measured using single items. In particular, instrumental support was measured by an item that focused on “helping with chores”—however, the concept of instrumental support encompasses many types of assistance, including financial support or help with transportation. Third, because the majority of the items in the ESSI measure the perceived availability of emotional support, overall support scores and emotional support scores were highly correlated (*r* = .96). Many studies do not separate the effects of social support by type, so future attention examining each subdomain will likely be informative. Fourth, the measure of perceived support did not include information about source of support, which did not allow us to investigate whether family support might be more strongly associated with CVD morbidity and mortality than other sources of support among racial/ethnic minoritized groups than among White individuals. In general, it may be advisable to develop and utilize a measure of perceived support based on the data and experiences of a more racially and ethnically diverse group of participants.

## Conclusion

In the Multi-Ethnic Study of Atherosclerosis, we observed that Black, Hispanic/Latino, and Chinese participants reported different levels of various types of support compared to White participants. Despite these differences, perceived informational support was associated with decreased risk of CVD morbidity to a similar degree in all racial/ethnic groups. These findings suggest that future research should disaggregate different types of support to examine their effects on cardiovascular outcomes and investigate the reasons why other types of support may not be as strongly linked to cardiovascular health. Understanding which types of support are most beneficial will improve the effectiveness of interventions aimed at enhancing social support and advance cardiovascular health.

## Data Availability

Data from the Multi-Ethnic Study of Atherosclerosis is available by request via https://www.mesa-nhlbi.org/Publications.aspx.
